# Implementation of Double Immune Checkpoint Blockade Increases Response Rate to Induction Chemotherapy in Head and Neck Cancer

**DOI:** 10.3390/cancers13081959

**Published:** 2021-04-19

**Authors:** Sabine Semrau, Antoniu-Oreste Gostian, Maximilian Traxdorf, Markus Eckstein, Sandra Rutzner, Jens von der Grün, Thomas Illmer, Matthias Hautmann, Gunther Klautke, Simon Laban, Thomas Brunner, Bálint Tamaskovics, Benjamin Frey, Jian-Guo Zhou, Carol-Immanuel Geppert, Arndt Hartmann, Panagiotis Balermpas, Wilfried Budach, Udo Gaipl, Heinrich Iro, Rainer Fietkau, Markus Hecht

**Affiliations:** 1 Department of Radiation Oncology, Friedrich-Alexander-Universität Erlangen-Nürnberg, Erlangen, 91054 Bayern, Germany; sandra.rutzner@uk-erlangen.de (S.R.); benjamin.frey@uk-erlangen.de (B.F.); Jianguo.zhou@externuk-erlangen.de (J.-G.Z.); udo.gaipl@uk-erlangen.de (U.G.); rainer.fietkau@uk-erlangen.de (R.F.); markus.hecht@uk-erlangen.de (M.H.); 2 Department of Otorhinolaryngology-Head and Neck Surgery, Friedrich-Alexander-Universität Erlangen-Nürnberg, Erlangen, 91054 Bayern, Germany; antoniu-oreste.gostian@uk-erlangen.de (A.-O.G.); maximilian.traxdorf@uk-erlangen.de (M.T.); heinrich.iro@uk-erlangen.de (H.I.); 3 Institute of Pathology, Friedrich-Alexander-Universität Erlangen-Nürnberg, Erlangen, 91054 Bayern, Germany; markus.eckstein@uk-erlangen.de (M.E.); carol-immanuel.geppert@uk-erlangen.de (C.-I.G.); arndt.hartmann@uk-erlangen.de (A.H.); 4 Department of Radiotherapy and Oncology, Goethe University Frankfurt, Frankfurt am Main, 60323 Hessen, Germany; jensvonderg@kgu.de (J.v.d.G.); Panagiotis.Balermpas@usz.ch (P.B.); 5 Medical Oncology Clinic Dresden Freiberg, Dresden, 01307 Sachsen, Germany; illmer@onkologie-dresden.net; 6 Department of Radiation Oncology, Universität Regensburg, Regensburg, 93040 Bayern, Germany; matthias-hautmann@klinik.uni-regensburg.de; 7 Department of Radiation Oncology, Chemnitz Hospital, Chemnitz, 09112 Sachsen, Germany; G.Klautke@skc.de; 8 Department of Otorhinolaryngology and Head & Neck Surgery, Universitätsklinikum Ulm, Ulm, 89081 Baden-Württemberg, Germany; simon.laban@uniklinik-ulm.de; 9 Department of Radiation Oncology, Otto von Guericke Universität Magdeburg, Sachsen-Anhalt, 39120 Magdeburg, Germany; thomas.brunner@med.ovgu.de; 10 Department of Radiation Oncology, Heinrich-Heine-Universität Düsseldorf, Düsseldorf, 40210 Nordrhein-Westfalen, Germany; Balint.Tamaskovics@med.uni-duesseldorf.de (B.T.); Wilfried.Budach@med.uni-duesseldorf.de (W.B.); 11 Department of Oncology, The Second Affiliated Hospital of Zunyi Medical University, Zunyi 563000, China

**Keywords:** combined modality therapy, head and neck neoplasms, induction therapy, double immune checkpoint inhibition, immunotherapy, HPV-positive OPSCC, organ preservation

## Abstract

**Simple Summary:**

The study compares the effects on complete remission rate (CR) of a single dose of durvalumab/tremelimumab immediately after a single-cycle platinum and docetaxel as part of induction therapy for a controlled trial in head and neck cancer with chemotherapy alone from a historical collective. The CR rate was 60.3% after induction chemoimmunotherapy (ICIT; induction chemotherapy plus double immune checkpoint blockade) compared with 40.3% after induction chemotherapy (IC) alone. Patients with HPV-positive oropharyngeal cancer may benefit the most from additive double checkpoint inhibition, which is presumably due to the higher amount of infiltrating immune cells. Patients older than 60 years without HPV-positive oropharyngeal cancer are unlikely to benefit.

**Abstract:**

To determine whether a single dose of double immune checkpoint blockade (induction chemoimmunotherapy (ICIT)) adds benefit to induction single-cycle platinum doublet (induction chemotherapy (IC)) in locally advanced head and neck squamous cell carcinoma (HNSCC), patients treated with cisplatin 30 mg/m^2^ d1-3 and docetaxel 75 mg/m^2^ d1 combined with durvalumab 1500 mg fixed dose d5 and tremelimumab 75 mg fixed dose d5 (ICIT) within the CheckRad-CD8 trial were compared with a retrospective cohort receiving the same chemotherapy (IC) without immunotherapy. The endpoint of this analysis was the complete response rate (CR). A total of 53 patients were treated with ICIT and 104 patients with IC only. CR rates were 60.3% for ICIT and 40.3% for IC (*p* = 0.018). In the total population (*n* = 157), the most important predictor to achieve a CR was treatment type (OR: 2.21 for ICIT vs. IC; *p* = 0.038, multivariate analysis). The most diverse effects in CR rates between ICIT and IC were observed in younger (age ≤ 60) patients with HPV-positive OPSCCs (82% vs. 33%, *p* = 0.176), while there was no difference in older patients without HPV-positive OPSCCs (53% vs. 48%). The analysis provides initial evidence that ICIT could result in higher CR rates than IC. Young patients with HPV-positive OPSCCs may have the greatest benefit from additional immune checkpoint inhibitors.

## 1. Introduction

Patients with locally advanced laryngeal carcinomas can be treated with induction chemotherapy (IC) to identify good responders for organ-preserving radiotherapy. These selected patients have similar tumor control rates compared with surgical resection [1]. IC has been adopted in hypopharyngeal carcinoma management as an organ preservation strategy [2]. However, IC does not improve overall survival compared with immediate chemoradiotherapy (CRT) and has been criticized for causing additional toxicity [3,4]. Therefore, instead of three cycles of chemotherapy, the administration of one cycle of induction chemotherapy followed by immediate tumor response assessment has become an established practice [5,6,7]. This approach ensures the administration of the full dose of CRT and achieves high organ preservation rates. However, there exists the limitation that single-cycle IC followed by CRT results in worsened swallowing function and prolonged percutaneous endoscopic gastrostomy (PEG) tube dependence despite organ preservation [8].

Consequently, chemotherapy-free definitive treatment is still a desired goal [9]. One strategy is to combine initial single-cycle IC with immune checkpoint blockade to select responders suitable for platinum-free radiotherapy. Evaluating the feasibility and effectiveness of such an induction chemoimmunotherapy (ICIT) approach is the subject of CheckRad-CD8, a single-arm multicenter phase II trial. The combination of anti-CTLA-4 and anti-PD-L1 in this trial should address two different immune checkpoints in tumor-related immunosuppression, which has been shown to be effective in malignant melanoma [10]. While anti-CTLA-4 eliminates the inhibition of CD4+ T cell activity in the lymph nodes, anti-PD-L1 is directed against the blockage of the CD8+ T cells in the tumor and in the peritumoral tissue [11,12]. Preliminary results indicate that this quadruple combination achieved a high complete response rate and good tolerability [13]. In 11% of the patients, there were higher-grade immune-related adverse events. The proportion of grade III and IV leukocytopenias was higher (43%) than that after chemotherapy in the historical cohort (21%), as well as the rate of infections (29% vs. 10%). However, the immunochemotherapy is feasible, and no treatment related death occurred [6,13].

The lack of comparison of ICIT with platinum doublet chemotherapy with docetaxel plus cisplatin or carboplatin, which is also regarded as effective, was a key comment on the preliminary results of the CheckRad-CD8 study.

Therefore, in the present study the efficacy of ICIT at a single study site was compared with that of IC (docetaxel plus platinum) in patients with locally advanced head and neck squamous cell carcinoma (HNSCC). The latter arm included a consecutive series of patients treated by the same study center and team.

## 2. Materials and Methods

### 2.1. Patients and Treatments

Data from 104 patients with locally advanced HNSCC who received single-cycle induction chemotherapy (docetaxel 75 mg/m^2^ on day 1 and cisplatin 30 mg/m^2^ or carboplatin AUC 1.5 on days 1–3) with subsequent tumor response assessment for further treatment decision-making regarding the feasibility of organ preservation from 2008 to 2020 were retrospectively collected and compared with prospective data from 53 patients who received ICIT consisting of the identical IC regimen plus double immune checkpoint blockade (PD-L1, CTLA-4) at a single CheckRad-CD8 trial center. Patients with locally advanced squamous cell carcinoma of the oral cavity, pharynx, and larynx were included, in which resection would have led to functional and organ loss. [5,6]. The CheckRad-CD8 trial also included patients whose tumors were considered nonresectable. The CheckRad-CD8 trial excluded patients who had systemic autoimmune or inflammatory disorders, active infections, or immunosuppressive medications [13]. For the use of carboplatin or cisplatin and docetaxel in both populations, the usual conditions applied to the kidneys and heart function [5,6]. The ICIT regimen consisted of durvalumab (1500 mg fixed dose) plus tremelimumab (75 mg fixed dose), both administered on day 5.

### 2.2. Clinical and Pathologic Response Assessment

Tumor response was assessed based on endoscopic examination of the tumor site, including a biopsy under general anesthesia carried out before treatment and on days 21 to 28 after the start of treatment. Complete response (CR) was defined as a tumor-free representative biopsy specimen from the former tumor region after induction treatment. In the case of ICIT, biopsy sites were located with positron emission tomography (PET) guidance before and after treatment. Thus, two specimens were collected from each ICIT patient, supported by PET guidance in 51 cases. In the IC group, endoscopic complete response (as determined by two examiners by panendoscopy) was used as a second criterion for complete response in case biopsy specimens were lacking or if a tumor-free biopsy was not clearly representative of the former tumor region. HPV association was analyzed via p16 immunohistochemistry.

### 2.3. Trial Oversight

The CheckRad-CD8 trial was registered with ClinicalTrials.gov (identifier: NCT03426657). The leading institutional review board at the Friedrich-Alexander-Universität Erlangen-Nürnberg (number: 131_18 Az) approved the trial. All patients gave written informed consent before the first study procedures were performed. All patients who received induction therapy agreed to the treatment concept. It was standard therapy. At the same time, they gave written consent to the subsequent compilation of data and scientific evaluation.

### 2.4. Statistical Analysis

Chi-square test and Fisher’s exact test were used to compare the frequencies of different variables in the two treatment groups. Because of the imbalances between the two groups, we used univariate and multivariate analysis to identify categorical predictive parameters for pCR in the whole population. In the multivariable analysis (logistic regression), all factors with a univariable *p*-value of <0.1 were considered in order to identify independent prognostic factors for pCR. The hypothesis of correlation could not be rejected if *p* < 0.05. The chi-square test or the *t*-test for independent samples were used to comparatively assess frequencies of CR between ICIT vs. IC for different single parameters thereafter. Since no independent predictor variable for CR could be identified within the two treatment groups by multivariate analysis, a hierarchical cluster analysis of various frequently occurring patient or tumor characteristics was ultimately performed in the group of ICIT. Within these groups, the frequencies of CR were compared between ICIT and IC using Fisher’s exact test. The statistical analysis was carried out using IBM SPSS Statistics Version 24.

## 3. Results

### 3.1. Patients

ICIT and IC groups were comparable in terms of gender, age, and N stage distribution, but showed imbalances in T classification and in Union for International Cancer Control (UICC) stage. The ICIT group included more patients with higher-stage HNSCCs and contained more patients with HPV-positive Oropharyngeal Cancer (OPSCCs) (Table 1). The subgroup of patients without HPV-positive OPSCCs contained more subjects with poorly differentiated squamous cell carcinomas (Table 1).

### 3.2. Correlation of CR Rate with Patient, Tumor, and Treatment Characteristics in the Overall Sample

Univariate analysis showed an association between CR rate and the type of induction treatment (*p* = 0.018) for the total population (*n* = 157), the type of tumor (HPV-positive OPSCC versus all other tumor types, *p* = 0.041), and T classification in tendency (*p* = 0.09). Neither nodal involvement, UICC stage, tumor grade, patient age, gender, nor tumor location was a factor associated with an increased probability of complete remission. In contrast to this, the type of therapy, ICIT vs. IC (odds ratio (OR): 2.28), and the T-Stage (OR: 1.48) were independent factors by multivariate analysis (Table 2).

### 3.3. Response to ICIT vs. IC

Following ICIT, 32 of 53 patients (60.3%) had no detectable residual tumor in directed biopsy specimens from the former tumor site, 20 had residual tumor, and 1 had a nonevaluable sample. In the IC group (*n* = 104), 32 patients had no histologically detectable residual tumor in representative biopsy specimens examined by panendoscopy, and 10 had sufficient evidence of clinical complete response to dispense with biopsy, whereas 33 patients had biopsy-confirmed residual tumor in control panendoscopy specimens, and 29 had macroscopic tumor persistence but no histologic specimen. Hence, 42 of 104 (40.3%) patients in the IC group were classified as complete responders. More patients achieved complete remission after ICIT than after IC alone (*p* = 0.018). CR rates based on histology alone were 60.3% (*n* = 32/53) for ICIT and 49% (*n* = 32/65) for IC (*p* = 0.019).

### 3.4. IC vs. ICIT: CR Rates by Individual Patient, Tumor, and Treatment Characteristics

In view of the dominant influence of the type of treatment, CR was analyzed as a function of the type of induction therapy (see Table 3). Laryngeal cancer patients and patients with poorly differentiated tumors (non-HPV-positive OPSCCs) had the smallest differences in CR rates. In HPV-positive OPSCC patients, CR rates were obviously higher with ICIT (71.4%) than with IC (40.0%), without gaining statistical significance. No difference in CR rates between ICIT (53.1%) and IC (40.9%) was observed in patients with non-HPV-associated tumors. ICIT resulted in significantly higher CR rates in men (+23%) than in women (+5%) and in younger (+32.2%) versus older patients (+7.3%) and in higher tumor stages, as shown by univariate analysis (*p* < 0.05).

### 3.5. CR Rates for IC vs. ICIT in Patient Subgroups with Identical Characteristics

**Figure 1:**
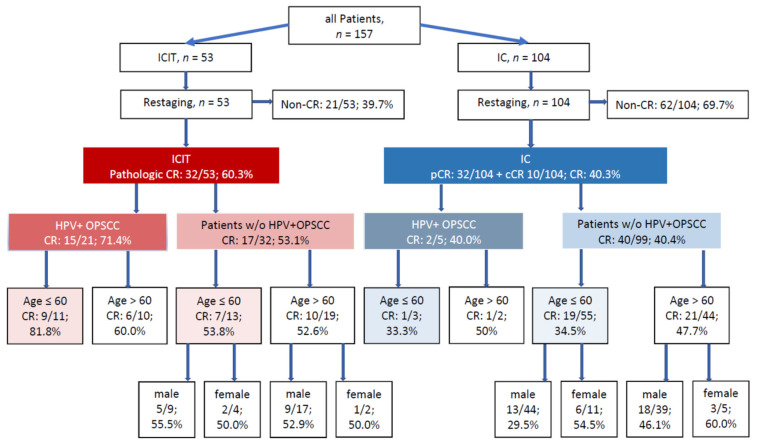
Frequent disease constellations and CR rates after ICIT versus IC in patients identified as having identical characteristics by hierarchical cluster analysis (pCR: biopsy without tumor, cCR: clinical complete response).

Hierarchical cluster analysis, including parameters with the greatest differences in CR rate (age ≤60 vs. >60, gender, HPV-associated OPSCC) showed four main subgroups within the ICIT group: patients with and without HPV-positive OPSCCs and younger (age ≤60) and older patients (age >60). Because gender played a subordinate role, the HPV-positive OPSCC subgroup was not further divided by sex due to the small sample size. CR rates for the two treatments and for the respective subgroups formed by hierarchical cluster analysis are shown in Figure 1. No statistically significant differences in CR rates were found between the groups with identical disease constellations. However, younger HPV-positive OPSCC patients tended to have the highest overall CR rates and the biggest differences in CR rates between ICIT and IC (81.8% vs. 33.3%, *p* = 0.176). In younger patients with HPV-negative OPSCCs or HPV-negative tumors in other sites, CR rates were considerably higher after ICIT (53.8%) than after IC (34.5%, *p* = 0.166), especially in men (55.5% vs. 29.5% for ICIT vs. IC, *p* = 0.133). There was no difference in CR rates between ICIT (52.6%) and IC (47.7%) in older patients without HPV-positive OPSCCs (*p* = 0.788).

## 4. Discussion

Immune checkpoint inhibitor therapy utilizing monoclonal antibodies directed against programmed cell death-1 (PD-1) and its ligand 1 (PD-L1) to target tumor-specific immune tolerance has become an established treatment modality for head and neck cancer patients with recurrent or metastatic HNSCC [14,15].

To our knowledge, this is the first study examining the additive, early treatment effect of combining a single dose of double immune checkpoint blockade to single-cycle induction chemotherapy in HNSCC. This work compares response data obtained with quadruple combination therapy with short-term response data obtained using the identical IC regimen in different patient groups.

In the ICIT group, 60% of the patients achieved biopsy-confirmed complete response after only one cycle of treatment. Compared with ICIT, IC alone resulted in an inferior CR rate (40%) in our study population. It should be noted that the proportion of biopsies, resection specimens, and histologically examined organs after laryngectomy in the IC group was 62%. This is much higher than the rates reported in many randomized trials. In a Spanish study of induction chemotherapy followed by chemoradiotherapy, for example, only 27% of the specimens were examined by histology, and the pCR rate was extrapolated to be 42% [16]. In a French study on the value of adding docetaxel to the induction chemotherapy regimen, biopsies were performed in 50% of the patients after three cycles of induction chemotherapy with TPF (docetaxel, carboplatin, and fluorouracil), and the investigators found that 62% of the biopsies were still tumor-free [17]. Another phase II study of TPF induction chemotherapy reported a pathologic complete response rate of 33% [18]. This and the complete response rates based on CT imaging observed after TPF was in the range of 17% to 43% in phase III studies [16,17,19,20,21]. Thus, induction chemoimmunotherapy resulted in at least similar CR rates compared with induction chemotherapy, which consisted of three cycles of TPF in the aforementioned comparative studies, but higher rates compared with one cycle of TP (docetaxel + cisplatin) in the present study. The fact that the results of the present study are based on biopsy samples alone is certainly subject to criticism, but in almost all cases, the specimens were taken from areas that were metabolically active identified by FDG-PET/CT before and after induction therapy.

The additional benefit of double immune checkpoint blockade with anti-CTLA-4 was not addressed in the present study. However, efficacy results vary for the combination of anti-CTLA-4 and anti-PD-L1 in head and neck cancer. Investigators in the EAGLE study of patients with locally recurrent or metastatic HNSCC reported an objective response rate (ORR) of only 17.9% for durvalumab and 18.2% for durvalumab plus tremelimumab and no survival benefit [22]. The CONDOR phase II trial likewise reported a low overall ORR for durvalumab (9.2%) and tremelimumab plus durvalumab (7.8%) and found no additive effect on the clinical response rate [23]. In the IMCISION trial of neoadjuvant nivolumab with or without ipilimumab prior to surgery (6 patients per group), 17% of the patients achieved near-complete pathologic response with nivolumab alone compared with 33% with nivolumab + ipilimumab [24]. Taken together, a benefit from additional CTLA-4 blockade has not been demonstrated so far, and the results of the studies raise a second question about any difference in the effectiveness of anti-PD-1 versus anti-PD-L1 in combination with chemotherapy.

Regarding PD-1 inhibitor monotherapy, a phase II clinical trial reported a pathologic complete response rate of 3.6% in HNSCC patients treated with one dose of neoadjuvant pembrolizumab monotherapy [25]. Of the 28 patients treated in the CIAO trial, 2 (8%) achieved a complete response after two cycles of neoadjuvant durvalumab with or without tremelimumab [26]. In summary, it can be concluded that PD-1/PD-L1 axis inhibition alone without chemotherapy does not result in a high response rate.

Compared with immunotherapy alone, we observed a significantly higher rate of complete remissions following ICIT, which suggests an additive effect. However, it is unclear whether distinct forms of chemotherapy or the timing of chemotherapy and immunotherapy have an additional catalytic effect on immunotherapy. In the KEYNOTE-048 study, investigators could not demonstrate any effect of combining simultaneous pembrolizumab with chemotherapy on the imaging-based response rate compared with chemotherapy alone: a response rate of 36.3% obtained with 5-fluorouracil plus cisplatin did not increase (35.6%) after the addition of pembrolizumab, but remission lasted longer [15]. A very high rate of CR of 100% was observed in the CIAO trial for 3 patients who received chemotherapy after immune checkpoint blockade [26]. In addition to the chronological sequence, the type of chemotherapy probably plays an essential role. A second evaluation of the TPextreme trial showed a better survival by 20 months if immunotherapy was used after taxane-based first-line chemotherapy (TPCet) than if 5-FU based chemotherapy (CFCet) was used [27].

Preliminary evidence also indicated which patient groups benefitted from the addition of immune checkpoint blockade, as reflected by a high response rate. PD-L1 expression levels on tumor and immune cells were identified as prognostic markers [15,28]. However, the first analysis of CheckRad-CD8 data showed an association between CR and PD-L1 expression on immune cells, but not on tumor cells [13].

The preliminary findings of the present study show that the quadruple combination achieved very high CR rates, especially in relatively young patients with HPV-positive oropharyngeal carcinomas with 81.8%. The HAWK study also detected differences in CR rates between HPV-positive and HPV-negative OPSCC patients following durvalumab monotherapy: 29.4% vs. 10.9%, respectively [29]. However, even our very small group of patients with HPV-positive OPSCCs achieved a CR rate of 40% after chemotherapy alone, which was slightly better than that after immunotherapy alone. In two early trials of induction chemotherapy followed by chemoradiotherapy, 64% of HPV-positive OPSCCs showed a clinical response (complete or partial) after one cycle of cisplatin or carboplatin plus fluorouracil [30], and 56% showed pCR after three cycles of IC with cisplatin, paclitaxel, and cetuximab [31]. Hence, it can be concluded again that a single cycle of induction chemoimmunotherapy is at least as effective as three cycles of cisplatin and taxane-containing chemotherapy in patients with HPV-positive OPSCCs and more effective than a single cycle of chemotherapy. One reason could be that the number of effector cells in HPV-associated tumors for the restitution of the immune response is higher than in non-HPV-associated tumors. A number of studies, summarized by Wodergem, show that the number of CD8 + T cells and regulatory T cells is increased in this tumor entity [32]. Consistent with that, another study observed that the stronger tumor response in HPV-positive OPSCC is due to a stronger immune response in HPV-driven OPSCC, associated with increased levels of tumor-infiltrating lymphocytes [33]. The CheckRad-CD8 study confirmed this: CD8+ cell densities were higher in HPV-positive OPSCCs than in tumors in other locations or HPV-negative once [13,34].

There are limitations of this trial: First of all, the retrospective character of the comparison; second, the largely heterogeneous groups with unequal and relatively small cohorts; and finally, the uncertainty of evaluating response rates in nonsurgically treated patients. In addition, the results of radioimmunotherapy after induction therapy conceived as definitive therapy followed by the consolidating 1-year immunotherapy in the CheckRad-CD8 trial are not yet mature, so the effectiveness and toxicity relevant for the patient compared with chemoradiotherapy have to be discussed at a later point in time. However, this is the first study comparing a “classic” induction chemotherapy with a quadruple immunochemotherapy as induction for head and neck cancer.

## 5. Conclusions

In conclusion, the addition of immune checkpoint inhibitors might increase the effectiveness of the induction scheme. However, different groups of patients may benefit to different degrees. Patients ≤60 years with HPV-positive OPSCCs could benefit the most, and older patients without HPV-positive OPSCC may have no advantage at all.

## Figures and Tables

**Table 1:** Patient characteristics (IC: induction chemotherapy; ICIT: induction chemoimmunotherapy; UICC: Union for International Cancer Control; OPSCC: Oropharyngeal Cancer; * Fischer’s exact test: ** *t*-test for independent samples).

Parameter	IC		ICIT		*p*-Value
	*n*	%	*n*	%	
Number	104		53		
Gender					
Male	84	80.8	45	84.9	*p* = 0.522
Female	20	19.2	8	15.1	
Median age (range)	58 (35–78)		61(38–78)		*p* = 0.27 **
T stage					*p* = 0.00001
1	1	0.9	2	3.8	
2	35	33.7	7	13.2	
3	42	40.4	10	18.9	
4	26	25.0	34	64.2	
N stage					*p* = 0.803
0	32	30.7	14	26.4	
1	17	16.3	10	18.9	
2a	1	1.0	2	3.8	
2b	26	25.0	13	24.5	
2c	27	26.0	14	26.4	
3	1	1.0	0	0	
UICC stage (7th Edition)					*p* = 0.045
2	12	11.5	0	0	
3	23	22.1	10	18.9	
4	69	66.4	43	81.1	
Grading					*p* = 0.016
1	4	3.8	0	0	
2	49	47.2	8	15.1	
3	46	44.2	24	45.3	
HPV-associated OPSCC					*p* = 0.000 *
No	99	95.2	32	60.4	
Yes	5	4.8	21	39.6	
Tumor location					*p* = 0.000
Oral cavity/oropharynx	18	17.3	33	62.3	
Hypopharynx	42	40.4	11	20.8	
Larynx	44	42.3	9	17.0	

**Table 2:** Uni- and multivariate analysis of different factors for achieving a complete remission in the whole population (CR: complete response, OR: odds ratio, CI: confidence interval, OCOP: oral cavity/oropharynx; HP: hypopharynx, L: larynx).

Prognostic Factor					Univariable	Multivariable		
		*n*	CR	%	*p*-Value	OR	CI	*p*-Value
Gender	Male	129	61	47	0.93			
	Female	28	13	46				
Age	<60 years	82	36	44	0.39			
	≥60 years	75	38	51				
T stage	1/2	45	26	58	0.09	1.48	1.02	2.150.037
	3/4	48	112	43				
UICC stage	II/III	45	23	51	0.52			
	IV	112	51	46				
Grading	1	4	2	50	0.23			
	2	57	20	35				
	3	70	35	50				
HPV-associated OPSCC	yes	26	17	65	0.041			0.368
	no	131	57	43				
Tumor location	OCOP	51	26	51	0.741			
	HP	53	23	43				
	L	53	25	47				
Therapy	ICIT	53	32	60	0.018	2.28	1.04–4.98	0.038
	IC	104	42	40				

**Table 3:** Complete response rates after induction IC versus ICIT as a function of patient, tumor, and treatment characteristics (* Fisher’s exact test).

Parameter	Complete Response Rate		*p*-Value
	ICIT	IC	
HPV-associated OPSCC			
Yes	71.4% (15/21)	40.0% (2/5)	0.145 *
No	53.1% (17/32)	40.9% (40/99)	0.208 *
Tumor location			
- Oral cavity, oropharynx	50.0% (6/12)	23.1% (3/13)	0.163 *
- Hypopharynx	63.6% (7/11)	38.1% (16/42)	0.119 *
- Larynx	44.4% (4/9)	47.7% (21/44)	0.575 *
Gender			
Male	62.2% (28/45)	39.2% (33/84)	0.013
Female	50.0% (4/8)	45.0% (9/20)	0.569 *
Age			
≤60 years	66.6% (16/24)	34.4% (20/58)	0.008
>60 years	55.1% (16/29)	47.8% (22/46)	0.351
T stage			
T1/2	77.7% (7/9)	52.7% (19/36)	0.164 *
T3/4	56.8% (25/44)	33.8% (23/68)	0.016
N stage			
N0–N2a	65.4% (17/26)	50.0% (25/50)	0.201
N2b/N3	55.5% (15/27)	31.5% (17/54)	0.037
UICC stage			
UICC Stage 2/3	70.0% (7/10)	45.7% (16/35)	0.160 *
UICC Stage 4	58.1% (25/43)	37.6% (26/69)	0.034
Grade			
G1		50.0% (2/4)	
G2	62.5% (5/8)	30.6% (15/49)	0.090
G3	50.0% (12/24)	50.0% (23/46)	1.00

## Data Availability

All source data relating to this manuscript are available upon request. Please contact sabine.semrau@uk-erlangen.de.
